# Competitive Cell Death Interactions in Pulmonary Infection: Host Modulation Versus Pathogen Manipulation

**DOI:** 10.3389/fimmu.2020.00814

**Published:** 2020-05-19

**Authors:** Ethan S. FitzGerald, Nivea F. Luz, Amanda M. Jamieson

**Affiliations:** Division of Biology and Medicine, Department of Molecular Microbiology and Immunology, Brown University, Providence, RI, United States

**Keywords:** bacterial pathogen, regulated cell death, lung, macrophages and neutrophils, pulmonary epithelium

## Abstract

In the context of pulmonary infection, both hosts and pathogens have evolved a multitude of mechanisms to regulate the process of host cell death. The host aims to rapidly induce an inflammatory response at the site of infection, promote pathogen clearance, quickly resolve inflammation, and return to tissue homeostasis. The appropriate modulation of cell death in respiratory epithelial cells and pulmonary immune cells is central in the execution of all these processes. Cell death can be either inflammatory or anti-inflammatory depending on regulated cell death (RCD) modality triggered and the infection context. In addition, diverse bacterial pathogens have evolved many means to manipulate host cell death to increase bacterial survival and spread. The multitude of ways that hosts and bacteria engage in a molecular tug of war to modulate cell death dynamics during infection emphasizes its relevance in host responses and pathogen virulence at the host pathogen interface. This narrative review outlines several current lines of research characterizing bacterial pathogen manipulation of host cell death pathways in the lung. We postulate that understanding these interactions and the dynamics of intracellular and extracellular bacteria RCD manipulation, may lead to novel therapeutic approaches for the treatment of intractable respiratory infections.

## Introduction

### Pulmonary Immune Response to Infection Overview

At homeostasis, the pulmonary system remains a tightly regulated environment with resident cell types performing highly specialized functions to maintain a pathogen-free space with unimpeded respiratory function. The immune response to respiratory pathogens requires carefully regulated processes to prevent uncontrolled pathogen replication and lethal tissue destruction (1–3). Such tissue destruction may be mediated by the pathogen virulence factors, the host response, or a combination of the two. Limiting such pathology requires precise orchestration of tissue cells, tissue resident immune cells, and infiltrating immune cells when responding to infections. Constant coordination between these cell types is orchestrated by secreted and intracellular signals, many of which are generated as byproducts or effectors of regulated cell death (RCD). Both tissue cells and immune cells undergo RCD processes during infections, with dysregulation of these complex RCD signaling networks often underlying the pathogenesis of pulmonary infectious disease. Below we provide a brief primer on the innate immune defenses of the lung and their status during homeostasis, before exploring the interplay of RCD signaling at the interface of bacterial pulmonary pathogens and host immunity.

Respiratory epithelial cells form a barrier between the outside environment and sterile zones in the body such as the blood (4). This is accomplished through a range of biophysical defenses, including the production of mucus by goblet or club cells and expulsion by ciliated epithelial cells (5). The composition of mucus also contains many antimicrobial peptides which impede bacterial translocation and effect bactericide (6). Furthermore, the physical barrier of tight cell-cell junctions formed between epithelial cells and underlying stroma is critical to barrier integrity (7). There is slow but constant turnover of respiratory epithelial cells via programmed cell death at homeostasis, with cells completely renewed every 30–50 days (8). Respiratory epithelial basal cells are stem cell-like cells which are long-lived and slowly dividing. They serve as progenitors for other subtypes of respiratory epithelial cells. The role and maintenance of the lung epithelium is reviewed extensively elsewhere (9).

Additionally, diverse lung resident innate immune cells patrol the lung (10). The majority of tissue resident phagocytes at steady state are alveolar macrophages (AMs). They reside in close proximity to respiratory epithelia and are responsible for immune surveillance. They recognize and are activated by pathogen-associated molecular patterns (PAMPs) and damage-associated molecular patterns (DAMPs). AMs serve as critical first responders that are required to both regulate signaling and carry out effector functions necessary to control bacterial infections of the lung. They are responsible for clearance of dead cells, debris, pathogens and inhaled particulates (10). In addition, AMs play important functions in maintaining homeostasis of the lung and promoting tissue repair through paracrine cell signaling (11). Other innate immune cells that are important in the lung immune response are interstitial macrophages and dendritic cells (DCs) (12). DCs in particular are sentinels throughout the lung epithelium and interstitium, where they serve as important early responders while helping to coordinate immunity to lung infections via antigen presentation (13). In addition to myeloid cells there are small numbers of lymphocytes that are found at steady state in the lung (14). If lung resident phagocytes cannot clear the infection, then other innate immune cells quickly infiltrate from circulation, and homing to secreted cytokines mediating immune cell recruitment (15). This includes neutrophils and inflammatory monocytes. Many of these resident and infiltrating innate immune cells are important replicative niches for distinct intracellular pulmonary pathogens. In addition, they are often targeted by cytocidal virulence factors from extracellular and intracellular pathogens, given their importance in initiating the sterilizing immune response. The pulmonary immune response is reviewed in greater detail elsewhere (10–15).

Respiratory epithelial cells and lung resident immune cells perform immune surveillance with a diverse repertoire of pattern recognition receptors (PRRs), such as Toll-like receptors (TLR), C-type lectin receptors (CLR), cytoplasmic retinoic acid-inducible gene-I-like receptors (RLR), and NOD-like receptors (NLR) (16). These PRRs are responsible for the detection of microbes in the respiratory system via the binding of PAMPs and DAMPs (17). Downstream signaling coordinated by PAMP/DAMP sensing cells includes the induction of bactericidal effector functions in immune cells such as reactive oxygen species (ROS) production, phagocytosis, or the secretion of mucus and bactericidal proteins in epithelial cells (18). PAMP-sensing cells that become overburdened by pathogens will also induce RCD processes (19). This can range from quiet apoptotic cell death to restrict pathogens within apoptotic bodies, or a pro-inflammatory pyroptotic cell death driving immune activity. Phagocytes responsible for engulfing pathogens also engulf dead or dying host cells via efferocytosis (20). Finally, upon clearing the infection, respiratory epithelial cells and immune cells in the lung coordinate the resolution of the inflammatory response and transition efforts from bacterial clearance to repair and remodeling in order to restore pulmonary homeostasis (21, 22).

### Cell Death Mechanisms

**FIGURE 1:**
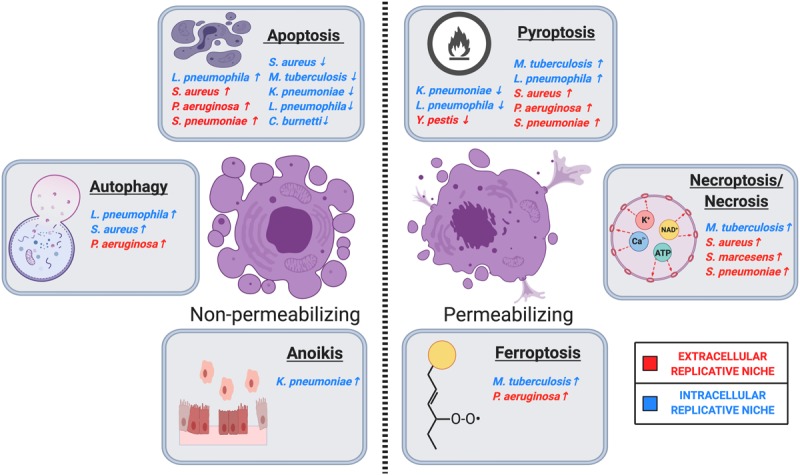
Regulated cell death in host-pulmonary bacterial interactions. Major cell death pathways are shown in the figure according to their membrane permeabilization status. Pulmonary bacteria induce or inhibit host cell death through several distinct modalities, including apoptosis, autophagy, anoikis, ferroptosis, necrosis/necroptosis, and pyroptosis. ↑ Indicates that the bacteria induces the indicated cell death pathway, while ↓ indicates that the bacteria inhibits the indicated cell death pathway. Intracellular or extracellular bacteria are labeled according to the legend. Image created with BioRender.com.

Cell death is intricately connected with life in multicellular organisms. The balance between cell death, proliferation, and differentiation is crucial for the maintenance of tissue homeostasis, and particularly in response to infectious disease. RCD is essential for many physiological processes, including cancer, neurodegeneration, autoimmune diseases, and response to infection (23, 24). This narrative review focuses specifically on the types of cell death that are modulated by bacteria during pulmonary infection that impact infection pathogenesis. All the cell types described above that are essential to the maintenance of a healthy lung are also targets of infection by bacterial pathogens. After infection, various types of RCD can be triggered by the host in an effort to control the pathogen, or by the pathogen in an effort to manipulate the host response to promote bacterial fitness. This is particularly relevant in the pulmonary epithelium, which provides a barrier that separates the air-filled compartment of the respiratory system from the aqueous interstitial compartment (25). Phagocytes, which are often a target of pathogen infection, have also evolved many adaptations to orchestrate diverse RCD pathways to promote resolution of infections (19, 26). Below, we will provide brief overviews of the cell death pathways activated during the pulmonary bacterial infections described in this review. We categorize the cell death types as those that do not cause significant disruption of the cellular membrane (membrane non-permeabilizing), and those that cause a lytic and inflammatory form of cell death (membrane permeabilizing). We classify apoptosis, autophagy, and anoikis as generally non-permeabilizing, and pyroptosis, necroptosis, and ferroptosis as generally permeabilizing (Figure 1). This classification was largely informed by recommendations from the Nomenclature Committee on Cell Death (24). Classically, forms cell death which resulted in membrane rupture were classified as “necrosis” and were thought to occur through an unregulated process. However, many recent works have determined that lytic cell death often occurs via regulated processes dependent on molecular signal transduction. Contemporarily and in this review, the term necrosis is used to describe the morphological loss of membrane integrity occurring as a result of regulated and non-RCD. It is important to note that this review is not meant to be a review on all aspects of cell death, but only how bacterial pathogens interact with cell death pathways in the lung. The underlying mechanisms of RCD are well described in other literature, which we have cited throughout the manuscript for further reference. However, this section on cell death mechanisms should serve as a brief primer on the subject.

Apoptosis is a form of RCD characterized by a distinct morphological phenotype in which cells shrink and undergo nuclear condensation, form outer membrane blebs, and fragment into apoptotic bodies (27). Throughout this process, cells maintain cytoplasmic membrane integrity, which serves to limit inflammation mediated by DAMPs, prevent the dissemination of intracellular pathogens, and enable efficient efferocytic clearance and recycling of cytoplasmic contents by phagocytes (28). The canonical forms of apoptosis are differentiated based on the extracellular or intracellular nature of the stimulus inducing RCD (29). Extrinsic apoptosis is mediated by the activation of membrane-bound extracellular receptors such as Fas Death Receptor 4/5, or TNFR1 (30). These receptors bind to cytokine signaling molecules produced by epithelial cells and immune cells that are sensing proximal pathogens, DAMPs from infected cells, or their own intracellular infection (30). Activation of these receptors by their ligand results in the assembly of the death-inducing signaling complex (DISC) (31). DISC assembly serves to promote caspase-8 activity, which proteolytically activates many proteins that mark the cells as actively undergoing apoptosis such as cFLIP, as well as caspase-3 and caspase-7 (31). The activity of caspase-3 and -7 effect the terminal progression of apoptosis (32). Intrinsic apoptosis is initiated by the sensing of a broad range of intracellular damage and stressors (33). However, with regards to bacterial pulmonary infections, intrinsic apoptosis is commonly induced by intracellular infections, genomic damage mediated by reactive free radicals, ER stress, or inactivation of inhibitory signals suppressing apoptosis (24, 34). Intrinsic apoptosis is terminally effected by the induction of mitochondrial outer membrane permeabilization when the correct combination of pro-apoptotic regulators are activated and anti-apoptotic signals are suppressed (35). Many of these regulators are members of the BCL protein family (35). Intrinsic apoptosis is also marked by the activity of caspase-3 and -7; however, they are activated by caspase-9 rather than caspase-8 (36). Both extrinsic and intrinsic apoptosis play critical roles in the control of pulmonary infectious diseases mediated by cell death signaling.

Autophagy has an important homeostatic role, mediating the removal of dysfunctional or damaged organelles through the action of lysosomes, which effects the recycling of diverse cellular components. Autophagy contributes to the maintenance of cellular homeostasis as part of many cell autonomous stress responses, including nutrient and growth factor deprivation, oxidative stress or hypoxia, the restriction of intracellular pathogens, and the resolution of immune responses (37, 38). Autophagy takes place in a series of steps. Autophagy initiation happens under starvation conditions when the unc-51-like kinase 1 (ULK1) complex dissociates from mTORC1 and becomes activated to initiate the formation of a phagophore. This is followed by a nucleation step driven by the PI3K complex. Phagophore elongation is then mediated by the Atg5-Atg 12-Atg16L and LC3II-PE conjugates. This results in the formation of autophagosomes. The autophagosomes then fuse to the lysosomes to form autophagolysosomes. The content present within the autophagosome is then delivered into the lysosomes where it gets degraded by the lysosomal enzymes (39). However, unrestrained autophagy can result in cell death. Based on guidelines published by the Nomenclature Committee on Cell Death, autophagy-dependent cell death is a type of RCD that relies on components of the autophagic machinery and takes place when the induction of autophagy coincides with the induction of apoptosis (24). Autophagic responses can play a key role in host defense to bacterial infections of the lung by reducing pathogen burden, but some pulmonary pathogens have evolved adaptions to benefit from such autophagic host responses or impede its execution (38, 40).

Anoikis is a form of regulatory cell death resulting from loss of cell anchorage to the basal membrane (41). Anoikis results in the loss of microtubule organization and basal cell junctions required for adherence to the extracellular matrix (41). Cellular escape from anoikis signal transduction is critical for the establishment of metastatic tumors, and anoikis has been implicated in bacterial and viral infections targeting cell anchorage (42). Further work is required to characterize the induction of anoikis RCD in the context of infectious disease.

Pyroptosis is a form of pro-inflammatory RCD that triggers mature IL-1β production and membrane permeabilization via gasdermins, which are host-derived pore-forming proteins (43). Pyroptotic signaling is triggered by recognition of PAMPs by NLRs. The PAMP-mediated activation of NLRs induces the assembly of multi-protein complexes called inflammasomes (44). There are many sub-types of inflammasomes, carrying out varying responses to unique pathogens (45). PAMPs from a given pathogen can trigger multiple inflammasome species (45). Generally, an activated inflammasome carries out the proteolytic maturation of caspase-1, which processes gasdermins, IL-1β, and IL-18 into their mature/active forms (46). Mature gasdermin proteins rapidly assemble into multimeric pore complexes that insert into the cell’s cytoplasmic membrane, resulting in cytoplasmic efflux and membrane-permeabilizing cell death (47). IL-1β and IL-18 are cytokines released during this process which promote inflammation and immune cell recruitment/effector function (46). Outcomes of pyroptosis induction vary from infection to infection, with pyroptosis having the capacity to both promote pathogen clearance and/or drive tissue damage and immunopathology.

The term “necroptosis” was originally introduced by Yuan et al., to delineate a specific form of regulated necrosis that is triggered by death receptor ligation and blockage of caspase-8 (48). One of the most intensively investigated subroutines of regulated membrane-permeabilizing cell death is necroptosis. The molecular pathway of tumor necrosis factor alpha (TNF-α)-induced necroptosis involves activation of Receptor-interacting serine/threonine protein kinase 1 (RIPK1)/Receptor-interacting serine/threonine protein kinase 3 (RIPK3)/mixed lineage kinase domain-like (MLKL) (49). Necroptosis is negatively regulated by caspase-8 and inhibitor of apoptosis proteins (IAPs), with the inactivation of caspase-8 resulting in the phosphorylation and activation of RIPK1, RIPK3, and MLKL which complex together to form the necrosome. To date, the only known kinase that is capable of phosphorylating MLKL is RIPK3, likely to reduce spurious induction of necroptosis. After becoming phosphorylated, MLKL oligomerizes into a membrane pore structure and inserts itself into the cell membrane, resulting in cytoplasmic leak (49). The unique molecular structure of MLKL oligomers enabling it to bind lipid bilayers is required for its pore forming properties (50). Insertion of sufficient amounts of oligomerized MLKL ultimately effects necroptosis al through lytic cell death (50, 51). The execution of necroptosis results in the disruption of ion homeostasis and the release of molecules that are recognized as DAMPs when in the extracellular environment (52).

Ferroptosis is a membrane-permeabilizing RCD dependent on iron in which glutathione, oxidative stress and lipid hyper-peroxidation play crucial roles. Suppression of glutathione antioxidant defense via inactivation of glutathione peroxidase 4 (GPX4) drives the hyper-peroxidation of lipids (53). Enrichment of membranes with polyunsaturated fatty acids, such as arachidonic acid (AA) esterified in phosphatidylethanolamines (PE), provides possible substrates for lipid hydroperoxides (54). The accumulation of hyper-peroxidized lipids within the cell results in a membrane-permeabilizing form of lytic cell death.

### Role of Cell Death During Bacterial Lung Infection Overview

The precise regulation of programmed cell death by respiratory epithelial cells and immune cells is a key determinant of morbidity and mortality (Table 1, 55). Hosts rely on infected cells initiating the appropriate programmed cell death response to prompt responding immune cells and proximal epithelial cells to restrict infections. In addition, uninfected immune cells must avoid cell death to carry out their functions. However, due to the evolutionary competition underlying every host-pathogen relationship, pulmonary pathogens have evolved amazingly diverse means to disrupt or redirect host cell death signaling toward cell death modalities that are beneficial to bacterial fitness. We argue that the specific modalities which bacteria aim to induce are primarily determined by the need to maintain a hospitable replicative niche for the bacteria. As such, bacterial tropism can be considered as an organizing principle when classifying the different cell death modalities bacteria seek to induce in hosts. The citations in this review evidence the claim that pulmonary bacteria have focused on modulating aspects of host cell death to better mold a replicative niche within the lung (Table 1). Interestingly, bacteria rely on both suppression and induction of RCD to accomplish these ends. This narrative review focuses on pulmonary bacterial pathogens where RCD plays an important role in their disease pathogenesis.

**TABLE 1:** RCD directionality through diverse pulmonary bacterial infections.

**Bacteria**	**RCD direction**	**Host target**	**Bacterial effector**	**Cell type**
*P. aeruginosa*	Apoptosis – ↑	TNFR1 (caspase-8–caspase-3 axis activation)	Quorum-sensing (N-3-oxo-dodecanoyl) (67)	Neutrophils from C57BL/6 mice and *in vivo* murine model
	Intrinsic apoptosis – ↑	Caspase-9 and effector caspase-3	ExoS (58)	Epithelial cells
	Apoptosis – ↑	Mitochondrial acid sphingomyelinase	Pyocyaninman (72)	Neutrophil (*ex vivo*), Jurkat T cells and HL-60 neutrophils
	Ferroptosis – ↑	15-hydroperoxy-AA-PE (15-HOO-AA-PE)	Lipoxygenase (pLoxA) (69)	HBE (human bronchial epithelial cell line)
	Pyroptosis – ↑	NLRC4 inflammasome	Type III secretion system	Phagocytes (THP-1 macrophages)
	Autophagy – ↑	unfolded protein response (UPR)	Type VI secretion system (71)	Human epithelial cells
*S. marcescens*	Necroptosis – ↑	RIPK1, RIPK3, and MLKL	ShlA (51)	Macrophages, neutrophil (*in vivo* murine model)
	Necroptosis – ↑	RIPK1, RIPK3, and MLKL	Pore-forming toxin (75)	Mouse bronchial epithelial cells (*in vivo* murine model)
*S. pneumoniae*	Intrinsic Apoptotis – ↑	Genomic DNA	Pyruvate oxidase produced ROS (84)	A549 human alveolar epithelial cell line and *in vivo* murine models
	Necroptosis – ↑	Cytoplasmic membrane	Pneumolysin (54)	A549 Human Alveolar Epithelial cell line and *in vivo* murine models
	Pyroptosis – ↑	Diverse inflammasomes	S. pneumoniae PAMPs (90)	Epithelial cells and immune cells
*S. aureus*	Apoptosis – ↑	Cell membrane and other Structures	Enterotoxins and α-toxin (52)	Leukocytes
	Apoptosis – ↑	Deoxyadenosine	S nuclease and adenosine synthase (103)	U937 (human lung macrophage cell line)
	Necroptosis – ↑	Mixed lineage kinase-like protein (MLKL)	Phenol-soluble modulines (PSMs) (103)	Neutrophil (*in vivo* murine model)
	Necroptosis – ↑	RIPK1, RIPK3, and MLKL	Pore forming toxins (99)	Human peripheral blood neutrophils and mouse bone marrow neutrophil
	Pyroptosis – ↑	NLRP3	agr, hla, lukAB, and PSMs (93)	Neutrophil (*in vivo* murine model)

**↑ Extracellular bacteria ↑ | ↓ Intracellular bacteria ↓**				

*S. aureus*	Apoptosis – ↓	iNOS	Arginine deaminase (110)	16HBE14o- Cell Line
	Apoptosis – ↓	MCL-1 (116)	Unknown Effector	Human marrow derived macrophages and RAW264.7 cell line
	Autophagy – ↑	Unc-51–like autophagy-activating kinase 1	agr quorum-sensing system (113)	Epithelial cells
	Autophagy – ↓	MAPK14 (114)	Unknown Effector	NIH/3T3 and mouse embryonic fibroblasts
*M. tuberculosis*	Apoptosis – ↓	iNOS activity/TLR Signaling	Esx-1 effector EspR (119)	RAW264.7 murine macrophage cell line
	Apoptosis – ↓	iNOS activity/MAPK-P53 Signaling	Esx-1 effector MptpB (120)	RAW264.7 murine macrophage cell line
	Apoptosis – ↓	ROS production/jnk signaling	AcpM Acyl carrier protein (121)	Murine bone marrow derived macrophages
	Apoptosis – ↓	IL10 mediated expression of TNFR2	HSP60 (124)	Differentiated Thp-1 human macrophage cell line
	Apoptosis – ↓	PPARg – MCL-1 signaling	Mannosylated lipoarabinomannan (125)	Human primary alveolar and monocyte derived macrophages
	Necrosis – ↑	Cytoplasmic membrane/phosphotidyl serine	Esx-1 effector ESAT-6 (126)	Human primary Neutrophils
	Pyroptosis – ↑	Unknown Effector	RD1 locus expression of Esx-1 and ESAT-6 (128)	Differentiated Thp-1 human macrophage cell line
	Necroptosis – ↑	NAD + Depletion	Tuberculosis necrotizing toxin (TNT) (129)	Differentiated Thp-1 human macrophage cell line
	Necrosis – ↑	PGE2 synthesis	Phospholipase C (132)	Rat primary alveolar macrophages
	Ferroptosis – ↑	Gpx4 peroxidase activity (133)	Unknown Effector	Murine bone marrow-derived macrophages and human monocyte derived macrophages
*K. pneumoniae*	Apoptosis – ↓	Bax-BCL2	*K. pneumoniae* capsule components (137)	Human primary neutrophils
	Apoptosis – ↓	Flippase regulation of phosphotidyl serine (139)	Unknown Effector	Murine peritoneal macrophages and neutrophils and *in vivo* murine models
	Pyroptosis – ↑	Diverse inflammasomes	*K. pneumoniae* PAMPs (141)	Murine bone marrow-derived macrophages and *in vivo* murine models
	Anoikis – ↑	Microtubule disassembly via KATNAL1 and KATNB1	YtfL (142)	A549 human alveolar epithelial cell line and *in vivo* murine models
*Y. pestis*	Pyroptosis – ↓	NLR pattern recognition receptors	YopK (146)	Bone marrow derived-macrophages and *in vivo* murine models
	Pyroptosis – ↓	Caspase-1	YopM (148)	Bone marrow derived-macrophages and *in vivo* murine models
	Pyroptosis – ↓	IQGAP1 Caspase-1 scaffolding protein	YopM (149)	Bone marrow derived-macrophages and *in vivo* murine models
	Pyroptosis – ↓	Pyrin inflammasome	YopM (150)	Bone marrow derived macrophages and *in vivo* murine models
	Pyroptosis – ↓	TAK1 – IKK IL1B activity	YopJ (151)	Bone marrow derived-macrophages
	Necrosis – ↑	Gasdermin D	YopK (151)	Bone marrow derived-macrophages
	Extrinsic apoptosis – ↓	FasL	Plasminogen activator (Pla) (146)	A549 human alveolar epithelial cell line, Jurkat cells, and *in vivo* murine models
*C. burnetti*	Intrinsic apoptosis – ↓	p32 transcription factor/Importin-alpha 1	AnkG (155)	Murine bone marrow-derived dendridic cells, CHO, HEK293T, and MEF cell lines
	Intrinsic apoptosis – ↓	p32 transcription factor	AnkG (155)	Murine bone marrow-derived dendridic cells, HELA, and HEK293T cell lines
	Intrinsic apoptosis – ↓	PARP cleavage	CaeA/CaeB (154)	Differentiated Thp-1 human macrophage cell line
	Intrinsic apoptosis – ↓	Bcl-1 and Bcl-2	Type IV secretion effectors (158)	HeLa cell line
*L. pneumophila*	Parthanatos – ↑	PARP	RpsL (173)	Bone marrow-derived macrophages
	Pyroptosis – ↑	NAIP5/NLRC4 (Birc1e/IPAF)	FlaA (171)	Bone marrow-derived macrophages
	Apoptosis – ↓	Caspase 3/7	Lipophosphoglycans (166, 169)	HeLa cell line, HEK293T cell line, and murine bone marrow-derived dendridic cells
	Apoptosis – ↓	BNIP3, Bcl-rambo	SidF (168)	Bone marrow-derived macrophages and U937 cell line
	Apoptosis – ↓	NF-kb	LegK1 and LnaB (169)	Bone marrow-derived macrophages
	Apoptosis – ↓	Caspase 3, 8,9, and 1	Dot/Icm, strain dependent (170)	Bone marrow-derived macrophages
	Pyroptosis – ↑	Caspase-11	Dot/Icm flaA-independent (166)	Bone marrow-derived macrophages and *in vivo* murine models
	Autophagy – ↑	Atg7, Atg, and MDC	Dot/Icm (169)	Bone marrow-derived macrophages

*Diverse pulmonary bacteria are listed in the table along with the RCD that the host cells undergo, host protein targeted by the bacteria, the bacterial effector used to manipulate cell death, and the cell type the finding was observed in. Intracellular bacteria are listed above the red line, and extracellular bacteria are listed below. ↑ Indicates that the bacteria induce the indicated cell death pathway, while ↓ indicates that the bacteria inhibits the indicated cell death pathway.*

Much experimental work has been conducted to elucidate cell death pathways implicated in the pathogenesis of pneumonia caused by diverse etiological agents. The application of small molecule inhibitors of key signal transducers for RCD pathways has shown that bacterial manipulation of RCD is often necessary for their survival *in vivo.* While there is much diversity in how pathogens manipulate RCD, we suggest that pathogens can be categorized based on: (1) intracellular or extracellular bacterial tropism and (2) whether pathogens can be regarded as inducers or suppressors of the inflammatory response. Briefly, we find that intracellular pathogens tend to manipulate RCD to promote the maintenance of the intracellular niche. Intracellular pathogens that induce the inflammatory response and immune cell recruitment rely on membrane-permeabilizing cell death to release bacteria from infected cells, rather than having them sequestered in membrane integral apoptotic bodies. Intracellular pathogens that suppress the inflammatory response seek to establish minimally immunogenic and chronic infections that evade recognition and clearance by the immune system. Many intracellular pathogens have evolved the ability to suppress RCD signal transduction by directly binding and inhibiting host factors.

Bacteria with extracellular tropism tend to aggravate the inflammatory response to promote tissue damage that speeds bacterial dissemination from the lung and releases crucial cytoplasmic nutrients into the comparatively nutrient poor extracellular space. They suppress the activity of immune effector cells and destroy epithelial barrier integrity by driving RCD through the secretion of toxins and other cytotoxic agents. Recent findings have determined that pore-forming toxins expressed by many pulmonary pathogens such as *Serratia marcescens*, *Staphylococcus aureus*, and *Streptococcus pneumoniae* stimulate necroptotic programmed cell death (56). Recombinant pore-forming toxins and bacteria-synthesized pore-forming toxins have been shown to induce necroptosis in both alveolar epithelial cells and in AMs, due to cytoplasmic dysbiosis resultant from loss of membrane integrity. These include ATP and metal ion efflux, mitochondrial damage, and ROS production. Necroptotic cell death can also be induced independent of PRR activation, through the activation of host proteins RIPK1, RIPK3, and MLKL, after sensing changes in the cytoplasmic environment such as ion and nutrient availability (57).

Given the centrality of RCD in determining pneumonia disease outcomes, it is clear that the pharmacologic or genetic manipulation of RCD during infection could represent a novel therapeutic strategy for the treatment of complicated or drug-resistant bacterial pneumonia (58). However, further study of the ways that pulmonary pathogens manipulate host RCD signaling during infection is required to design effective therapeutic strategies for validation. This review aims to provide a survey of pneumonia-causing bacterial manipulation of RCD and begin defining classifications of bacterial pulmonary pathogens based on their manipulation of RCD. By aggregating such information of diverse pathogens, trends regarding bacterial pathogenesis mechanisms can be elucidated to inform future work investigating bacterial manipulation of RCD and host-targeted therapeutic strategies.

## Pathogen-Specific Regulated Cell Death Responses

### Extracellularly Replicative Bacteria

#### Pseudomonas aeruginosa

*Pseudomonas aeruginosa* is an extracellular, Gram-negative pathogen (59). While considered primarily an extracellular pathogen, Bajmoczi et al., have reported that *P. aeruginosa* can be found in epithelial cells (60). However, we will focus on its primary extracellular tropism. *P. aeruginosa* opportunistically causes serious infections and is a leading cause of nosocomial pneumonia. It also contributes to morbidity and mortality due to respiratory failure and sepsis in immunocompromised patients, particularly in cystic fibrosis patients (59, 61). It is noteworthy that there has been a significant increase in the incidence of multi-drug resistant isolates of *P. aeruginosa* in hospital settings, making complementary therapeutic approaches to augment immune clearance and control RCD in epithelial and endothelial pulmonary tissue more essential (62).

The antibacterial host response initiated during *P. aeruginosa* infection is multifaceted and includes the activation of RCD pathways including apoptosis, necroptosis, and pyroptosis. *P. aeruginosa* induces endothelial cells to undergo apoptosis, through mechanisms that are partially dependent on the oxidative stress response (63). The production of ROS is classically known to be an essential mechanism for the immune cell mediated killing of *Pseudomonas* (64). Such apoptosis induction is likely a byproduct of these bactericidal activities. *P. aeruginosa*-infected endothelial cell death via apoptosis has been adapted to contribute host elimination of *P. aeruginosa* because apoptotic cells and associated bacteria are readily efferocytosed by professional phagocytes, particularly AMs (65). *P*. *aeruginosa* adhesion receptors fail to distinguish between adherence of apoptotic epithelial cells and epithelial cell apoptotic bodies, similarly allowing *P*. *aeruginosa* to be internalized through efferocytosis (65). However, due to the importance of endothelial barrier integrity, death and the elimination of infected cells from the endothelium lining can also provide a route for dissemination of *P. aeruginosa* to distal sites where metastatic infections can be established.

Given the restrictive effects of such epithelial or endothelial tissue cell apoptosis on these infections, it stands to reason that *P. aeruginosa* and other bacteria may have developed strategies to counteract this RCD. However, *P. aeruginosa*, as an extracellular pathogen, has mainly evolved protein effectors, which agonize cell death. This is consistent with the trend that extracellular bacteria prioritize activities that are detrimental to pulmonary barrier integrity as opposed to modulating cell death to inhibit bacterial clearance. For instance, *P. aeruginosa* derived LPS is sufficient to induce human alveolar morphological changes, inflammation, and apoptosis in A549 and human bronchial BEAS-2B cells (64). Exotoxins from the type III secretion system (T3SS), once in the cytoplasm of host epithelial cells, also have been shown to induce cell death by programmed necrosis or apoptosis, thus favoring disruption of epithelial barriers (66).

Some *P. aeruginosa* quorum-sensing metabolites also function to induce host immune cell death through cell surface lipid domain dissolution (67). Bacterial quorum-sensing auto-inducers are small chemicals released to control microbial community behaviors. Interestingly, one of these quorum sensing metabolites, N-(3-oxo-dodecanoyl)-L-homoserine lactone, was found to integrate into host cell membranes and induce RCD in responding immune cells through dissolution of the lipid bilayer. The effect of this membrane disruption resulted in host cells expelling tumor necrosis factor receptor 1 (TNFR1) associated with its membrane into the disordered lipid phase. There it trimerized into its active form and drove caspase-3–caspase-8-mediated apoptosis. Thus, *P. aeruginosa* gains a survival advantage by inducing RCD in responding leukocytes which suppresses bacterial clearance. Song et al., noted that the suppression of caspase activity effecting apoptotic RCD, was able to diminish pathogenicity by inhibiting this effect.

*Pseudomonas aeruginosa* also utilizes secreted exotoxins such as ExoS and ExoT, two homologous T3SS virulence factors that induce apoptosis in target host epithelial cells through toxin associated stress responses relating to cytoskeletal regulation (68). A recent work showed that T3SS also activates the cytosolic nucleotide-binding domain, leucine-rich repeat-containing caspase activation and recruitment domain CARD_ containing 4 (NLRC4) inflammasome, which activates caspase-1 and induces gasdermin-mediated cytotoxicity and the release of mature IL-1β (68). ExoU-producing isolates of *P. aeruginosa* caused massive cell death *in vitro* in THP-1 human monocytes but drove minimal release of IL-1β. In contrast, those expressing T3SS but not ExoU induced caspase-1 activation and IL-1β release, the level of which was correlated with cytotoxicity. Both effects were prevented by a specific caspase-1 inhibitor; however, further forms of cell death have not been examined in this model. Thus, T3SS cytotoxicity is mediated partially through the modification of inflammasome regulated cytokine production for *P. aeruginosa* clinical isolates that do not express ExoU.

*Pseudomonas aeruginosa* have also been found to induce ferroptosis, which is a cell death program executed via selective oxidation of arachidonic acid–phosphatidylethanolamines (AA-PE) by 15-lipoxygenases (69). *P. aeruginosa* can express lipoxygenase (pLoxA), which oxidizes host AA-PE to 15-hydroperoxy-AA-PE (15-HOO-AA-PE), and triggers ferroptosis in human bronchial epithelial cells. A biofilm of *P. aeruginosa* is capable of inducing ferroptosis in human bronchial epithelial cells via enhanced expression of pLoxA and oxidation of host cell AA-PE to 15-HOO-AA-PE (69). To date, the majority of work on *P. aeruginosa*-phagocyte interactions has focused on apoptosis, and to a lesser extent, pro-inflammatory/membrane-permeabilizing forms of cell death, such as ferroptosis. Further research is required to determine whether ferroptosis induction is an effective strategy employed by *P. aeruginosa* to promote disruption of the epithelial barrier and immune-regulatory functions.

*Pseudomonas aeruginosa* also utilizes a type VI secretion system (T6SS) to secrete numerous virulence effectors that can both interfere with competing microbes and manipulate host cells (70). *P. aeruginosa* infection induces autophagy in epithelial cells and this response plays a vital role in clearing intracellular bacteria. In addition to epithelial cells, neutrophils also play a pivotal role in the host’s early acute defense against pulmonary *P. aeruginosa*. Pyocyanin is a membrane-permeable pigment that also functions as an exotoxin when released by *P. aeruginosa* by inducing neutrophil apoptosis (71). Pyocyanin interacts with components of the mitochondrial electron transport chain, driving the dysregulated release of ROS, activation of mitochondrial acid sphingomyelinase, synthesis of mitochondrial ceramide, and the release of pro-apoptotic cytochrome c from the intermembrane space (71). This mechanism is associated with neutrophil depletion, an event that can sensitize the host to *P. aeruginosa* infections and colonization, since neutrophils are key elements of the host defense against infection (71).

Summary – *P. aeruginosa* largely conforms to the expected behaviors of pulmonary extracellular bacterial pathogens by inducing RCD in tissue and immune cells, rather than suppressing restrictive RCD mechanisms. *Pseudomonas* utilizes the secretion of exotoxins to damage cellular structures and tissues while coordinating colony behavior. Toxins also act to suppress the activity of host immune cells. Like many pyrogenic infections, PAMP and DAMP signaling are necessary for bacterial clearance, but also drive tissue damages through the induction of stress responses. Therapeutic strategies augmenting the cytoprotection of immune cells through suppression of their apoptotic signaling while driving tissue cell efferocytic clearance and phagocytic activities, may serve as critical measures in the context of antibiotic-resistant nosocomial infections. Designing and validating such RCD modulating therapeutic strategies could significantly improve clinical options for immunosuppressed and cystic fibrosis patients who often suffer from these infections.

#### Serratia marcescens

*Serratia marcescens* is an opportunistic Gram-negative bacteria classified in the order Enterobacteriaceae and the family *Yersiniaceae*. *Serratia* species are not typical constituents of human microbiomes, more commonly residing in environmental niches such as fresh water, soil, and the fecal flora of many animals (72). However, *S. marcescens* has also been identified a nosocomial pathogen causing diseases including severe bacterial pneumonia, in part due to its ability to persist on abiotic substrates for extended periods and the emergence of antibiotic resistant strains (73).

Limited data exists characterizing Serratia infections in pulmonary model systems. For instance, *S. marcescens* was show to produce ShlA, a pore-forming toxin that effects macrophages by inducing necroptosis, damaging mitochondrial membranes, inducing ATP depletion via cytoplasmic leak, and driving ROS production (74). Recent work found that the activity of ShlA was dependant on its activity as a calcium channel disrupting Ca^2+^ ion homeostasis, and that the effects ShlA toxicity could be inhibited *in vitro* through the direct application of Ni^2+^ ions (75). In an *in vivo murine* model of hemorrhagic pneumonia caused by *S. marcescens*, inhibition of necroptosis via RIPK1 and MLKL inhibition resulted in decreased morbidity and mortality (56). A previous study has also shown that *S. marcescens* is able to induce apoptosis in human lung adenocarcinoma A549 cells (76). In addition, Krzymińska et al., reported that hospital-isolated strains of *S. marcescens* produce toxins that contribute to its virulence and are essentials for the bacteria to adhere and invade to epithelial cells and induce hemolysis, cytotoxicity, and apoptosis of human epithelial cells and macrophages (77).

While very few mechanistic details characterizing *S. marcescens* pathogenesis and host cell death modulation in pulmonary system are available, significant *in vitro* data illustrates how *S. marcescens* also targets host RCD signaling known to exacerbate pneumonia and manipulates RCD signaling that is useful for defense against pulmonary infections. Such RCD manipulation strategies likely play key roles in the pathogenesis of *S. marcescens* in the clinic. For instance, direct injection of *S. marcesens* into the hemolymph of silkworms agonized c-Jun NH2-terminal kinase signaling which eventually resulted in the caspase mediated apoptosis of blood cells (75). This effect was also observed during *in vitro* infection of mouse peritoneal macrophages (78). Using a transposon library for forward genetic screening, Ischii et al., found that apoptosis induction was inhibited with the loss of genes necessary for lipopolysaccharide and flagellin synthesis (78).

Additionally, work performed in a Chinese Hamster Ovary epithelial cells, HeLa cells, *Atg 5*+/+ Mouse Embryonic Fibroblasts, and T24 cells revealed that *S. marcescens* is capable of manipulating autophagy to establish an intracellular niche in diverse cell types (73). During *in vitro* infection, approximately 20% of bacteria were able to induce endocytic uptake in these cells and form intracellular replicative niches in Rab7 and LC3 positive vesicles in a Atg5 dependent manner. Despite these autophagic markers, *S. marcescens* containing vesicles failed to acidify and restrict bacterial growth (73). Fedrigo et al., hypothesized that cell wall components of *S. marcescens* may play a key role in the induction of this process. Such manipulation of epithelial cell autophagy in the pulmonary environment, could significantly agonize respiratory epithelial cell death due to the induction of intracellular stress responses that may help shield the bacteria from responding host immune cells and phagocytes. Further investigation of *S. marcescens*’ ability to manipulate autophagy in the context of pulmonary infection capacities is required.

*In vitro* data also suggests specific bacterial proteins and peptides that are able to manipulate RCD. For instance, non-pore forming toxin produced by *S. marcescens* was shown to induce cytolysis (79). Ectopic expression of *S. marcescens* PhlA was found to induce cytolysis in HeLa cells via the direct hydrolysis of phospholipids to lysophosphoylipids, which effected membrane permeabilization after they are incorporated into the cytoplasmic membrane (79). Hydrolyzed lysophospholipids generated by PlaA are capable of triggering both hemolysis and cytolysis. Another Serratia derived peptide, AT514 or serratamolide, was also found to strongly induce apoptosis in typically apoptosis resistant B cell chronic lymphocytic leukemia cells (80). Administration of the peptide to tumor cells cancerous B cells effected the induction of intrinsic apoptosis through the release of cytochrome c from mitochondria and the activation of caspase-9 and caspase-3 (80). AT514 was also found to inhibit the pro-survival signals of Bcl2, phosphatidylinositol-3 kinase, and protein kinase C. Also, the *S. marcescens* derived metabolic compound prodigiosin was found to agonize p53-induced apoptosis and suppress the activity of survivin in acute lymphoblastic leukemia. This is accomplished by driving the synthesis of ROS, that damage dsDNA, RNA, and other cell components (81). The observation of multiple apoptosis inducing compounds and cellular components produced by *S. marcescens*, lends credibility to the hypothesis that the induction of apoptosis aggravates *S. marcescens* pneumonia and other forms of nosocomial infections.

Summary – Generally, *S. marcescens* induces cell death to disrupt epithelial cells–extracellular matrix adhesions in airway epithelial cells, which allows for bacteria invasion into the submucosal tissues. Alternatively, when *S. marcescens* induces necroptosis through pore-forming toxins this leads to enhanced disease severity. Induction of cell death within cells of the immune system may contribute to the spread of infection and prolonged disease manifestation. One of the most catastrophic of *S. marcescens’* strategies is the induction of necroptosis in AMs. This not only abolishes a critical element of the early immune response, but also results in inflammation and tissue damage, which intensifies disease. Thus, targeting necroptosis may provide an important therapeutic strategy to block both cell death and inflammation for the treatment of human hemorrhagic pneumonia triggered by *S. marcescens*.

#### Streptococcus pneumoniae

*Streptococcus pneumoniae* is a Gram-positive bacteria commonly causing pulmonary infections with a preferred extracellular tropism (82). *S. pneumoniae* effects much of their virulence through direct killing of responding immune cells and respiratory epithelial cells via ROS production and the secretion of pore-forming toxins. While these effector functions do not directly manipulate or suppress host proteins regulating cell death to promote their survival, their activity has been shown to drive several host RCD pathways which underlie much of the pathogenesis of *S. pneumoniae* infection and impede host clearance efforts.

The *S. pneumoniae* enzyme SpxB, a pyruvate oxidase, acts upon bacterial intracellular pyruvate to generate H_2_0_2_ ROS that are secreted into the pulmonary microenvironment. Elevated ROS has been shown to induce double-strand breaks in the host’s genome and result in a p53-dependent apoptotic cell death that can exacerbate tissue damage (83). However, this induction of apoptosis can be pro-resolving in the context of *S. pneumoniae* that have already been engulfed by phagocytes, particularly macrophages. In murine infection models, inhibiting the activity of caspases that cause apoptosis was shown to increase pathogen burden (84). Apoptotic death of some AMs during *S. pneumoniae* infection has also been proposed to limit hyper-inflammatory damage associated with severe bacterial infection and promote efferocytic clearance of bacteria-laden phagocytes. It has recently been observed that inhibition of TNF-induced AM apoptosis via TNF-related apoptosis-inducing ligand (TRAIL) knockout significantly inhibited bacterial clearance and survival in a murine *S. pneumoniae* infection model. The inhibition of TNF-induced apoptosis drove AMs toward a necrotic cell death phenotype, which was able to be rescued through TRAIL add back and an application of anti-DR5 antibodies to drive apoptosis without an upstream TNF signal. They also determined that dying neutrophils served as a major source of TRAIL, and hypothesized that an inability to induce macrophage apoptosis during early pneumococcal infection may explain the severe pathology of neutropenic hosts during pulmonary bacterial infection (85). Other factors inhibiting macrophage apoptosis, such as host-derived MIF or bacterial PcpA, have also been shown to be detrimental to the host during pneumococcal infection (86, 87). For instance, recent research has demonstrated that AM-specific expression of the anti-apoptotic protein MCL-1 in mice inhibited the cells’ capacity to kill phagocytosed *S. pneumoniae*. Researchers generated a transgenic mouse line constitutively overexpressing human MCL-1 protein in AMs. The researchers found that wild-type AMs would phagocytose *S. pneumoniae* and carry out killing through the phagolysosome. However, prolonged uptake of bacteria over 12 h eventually overwhelms the phagocytic clearance pathway, resulting in the activation of macrophage apoptosis which drives the release of mitochondrial-derived ROS and nitric oxide production to enhance killing of intracellular bacteria. While MCL-1 overexpressing AMs were able to successfully uptake *S. pneumoniae* and activate nitric oxide synthesis after becoming overwhelmed with intracellular bacteria, they were unable to release mitochondrial-derived ROS to kill bacteria (88).

Many independent research groups have demonstrated the induction of regulated membrane-permeabilizing cell death via pyroptotic or necroptotic signaling to be detrimental to the host response to pneumococcal pneumonia, primarily due to the hyper-inflammatory response these modes of cell death promote. For instance, recent work demonstrated that nucleotide-binding domain, leucine-rich repeat-containing, and pyrin domain-containing 3 (NLRP3) and apoptosis-associated speck-like protein containing a caspase recruitment domain (ASC) knockout mice had improved host defense in a lethal infection model with *S. pneumoniae* (89). The inhibition of pyroptosis is thought to improve host defense to pneumococcal pneumoniae by suppressing hyper-inflammation that is detrimental to pulmonary barrier integrity without inhibiting bacterial clearance by phagocytes.

Like many other pulmonary bacterial pathogens *S. pneumoniae* produces pore-forming toxins leading to necroptosis (56). The pore-forming toxin pneumolysin (Ply) is one of the most well characterized virulence factors produced by *S. pneumoniae*. *S. pneumoniae* Ply (like many bacterial pore-forming toxins) drives necroptotic cell death signaling in diverse cell types, including alveolar epithelial cells and macrophages. *S. pneumoniae* species that induce lower degrees of NF-kB activity have been associated with more severe pneumonia (90). Lower NF-kB activity has also been shown to drive macrophages away from an active state focused on bacterial clearance and toward pro-inflammatory necroptosis (90).

Summary – *S. pneumoniae* are bacteria that prefer to replicate extracellularly on the surface of respiratory epithelium. They primarily induce host cell death in responding immune cells and respiratory epithelial cells through the secretion of protein or chemical toxins that damage cellular structures. These include pneumolysin, which damage cell membranes by forming pores, and bacteria-synthesized ROS, which induce genomic damage to drive apoptosis. In the context of engulfing macrophages, bacterial surface proteins have been shown to inhibit pro-resolving macrophage apoptosis. Host-directed therapeutic strategies augmenting phagocyte or respiratory epithelial cell resistance to the cytocidal effect of *S. pneumoniae*-derived protein or chemical toxins may help ameliorate the burden of pneumococcal pneumonia, in conjunction with current antibiotic therapies and vaccination strategies. These could include inhibitors of membrane permeabilizing RCD or strategies to limit the impact of ROS.

#### Staphylococcus aureus

*Staphylococcus aureus* has been classically identified as extracellular pyrogenic bacteria, but it has also been recognized to have facultative intracellular tropism during infection (91). Below we explore the behavior of *Staphylococci* as an extracellular bacteria in the pulmonary space, and we will discuss the intracellular ramifications in the section “Intracellularly Replicative Bacteria.” An array of the *S. aureus* proteins function as virulence factors that worsen the pathogenicity of infection. For instance, *S. aureus* produces potent toxins, such as staphylococcal enterotoxins (SEs) and α-toxin (α-hemolysin), which have been shown to induce biological membranes disruption (92). In spite of several toxin-mediated cytotoxic properties, only α-toxin (α-hemolysin) and Panton-Valentine leukocidin (PVL) have been reported to promote exhibit pro-apoptosis-like death in host cells (93). Apoptosis induced by membrane-damaging toxins is characterized by caspase-3 and caspase-9 activation, as well as activation of intrinsic mitochondrial-mediated apoptotic signaling pathway (93, 94). This apoptosis induction is primarily associated with host phagocytes and induces stress responses. By targeting these important immune cells, the host immune response is suppressed and this allows *S. aureus* to maintain extracellular colonization of host tissues in necrotizing biofilms.

Extracellular *S. aureus* also impart much of their characteristic necrotic tissue damage, via the expression of secreted toxins as a major mechanism to induce lung damage through necroptosis induction through RIPK1/RIPK3/MLKL (92). *S. aureus* mutants lacking pore-forming toxins, such as Psms, agr, and hla are less effective at inducing cytotoxicity in human and murine immune cells. Host directed perturbations of necroptosis by inhibition of either RIPK1 or MLKL were also shown to decrease *S. aureus*-mediated cytotoxicity in immune cells *ex vivo*, but this RCD activity may be pro-resolving *in vivo* (92). These secreted factors from extracellular *S. aureus* biofilms impart necrotic tissue damage by destroying both epithelial and endothelial cells in the lung, which also promotes severe lung barrier disruption and pulmonary edema (95). Also, secreted toxins will act as leukocidins to dampen the effectiveness of responding phagocytes by inducing RCD and disrupting phagocytic clearance activity (96).

These observations are characterized particularly well in neutrophils. Neutrophil recruitment and activity is necessary to eradicate *S. aureus* from the lung. In a mouse model of *S. aureus*-induced pneumonia, NLRC4-associated necroptosis was observed in infiltrating neutrophils. This RCD was shown to drive interleukin-18 (IL-18), which results in the suppression of IL-17A signaling from T cells. This suppression of IL-17A results in decreased neutrophil recruitment to the lung which in turn exacerbates *S. aureus* pneumonia by inhibiting successive waves of neutrophil recruitment required to clear the infection (97). The induction of neutrophil necroptosis also triggers the release of neutrophil extracellular traps (NETs), which typically restrict extracellular pathogens such as extracellular *S. aureus*. In neutrophils, MLKL translocates to the plasma membrane and binds phosphatidylinositol phosphates, which activates NADPH oxidase-derived ROS production to trigger the breakdown of the nuclear membrane and extrusion of bacteriostatic NETs (98). This process is known to require RIPK1, as RIPK1 inhibition or kinase deficiency inhibits NET generation by mouse and human neutrophils (98). 98 have also reported that peptidylarginine deiminase 4 (PAD4) is essential for anti-Staphylococcus innate immunity mediated by NET extrusion (27). It is also important to note that NET release can also be necroptosis independent (98). Alternative mechanisms of NET release include ligation of adhesion receptors, including CD11b, CD18, and CD15 or paracrine signaling downstream of lipopolysaccharide (LPS) detection (99, 100). It has also reported that the induction of necroptosis can limit excessive inflammation that worsens *S. aureus* infection, in part by limiting the expression of IL-1. MLKL knockout mice that were unable to execute necroptosis, were shown to redirect RCD to a pyroptotic and hyper-inflammatory cell death phenotype during *S. aureus* infection (101). The effect of MLKL activity and necroptosis in neutrophils was reported to be pro-resolving in this model.

However, *S. aureus* has been shown to subvert the host immune response mediated by neutrophil necroptosis and NET release by secreting nuclease and adenosine synthase A, which convert NETs to deoxyadenosine (dAdo). This also drives dATP formation that can induce caspase-3–dependent apoptotic RCD in phagocytes. Disruption of this signaling pathway rescues macrophages that had taken up dAdo from caspase-3–induced cell death (102). The induction of apoptosis in responding host immune cells, helps to promote the maintenance of the extracellular niche and the defense of *S. aureus* biofilms from the activity of innate immune phagocytes. The activity of *S. aureus* alpha toxin has also been found to suppress the efferocytic clearance of apoptotic phagocytes, particularly dead neutrophils by AMs *ex vivo* and *in vivo* (103). The lingering of apoptotic cells activity resulted in higher degrees of cell lysis, likely via secondary necrosis. This would tend to drive greater DAMP sensing in the pulmonary space during *in vivo* infection. This DAMP sensing may agonize pyroptosis induction, which has been associated with worse *S. aureus* infection outcomes. However, more research is required to elucidate these potential disease mechanisms.

Summary – Extracellular *S. aureus* causes acute pyrogenic infections in the lung by forming biofilms around colonies that induce severe tissue damage and impede phagocytic clearance. Tissue damage is imparted by the activity of extracellular toxins on tissue cells, which induce necroptotic cell death. PAMP and DAMP sensing also induces pyroptosis in tissue cells. The induction of these membrane permeabilizing RCDs in tissue cells release nutrients for the colony and diminish lung barrier and respiratory function. Dying tissue cells and sentinel cells recruit phagocytes which are similarly targeted by secreted toxins to impede their immune effector functions. Secondary metabolic effectors generated by *S. aureus* also trigger stress responses in immune cells resulting in apoptotic RCD and impaired function. The dual intracellular and extracellular tropisms of *S. aureus* make the design of host directed therapeutics very challenging. Suppression of the immune response to ameliorate tissue damage may promote bacterial cell growth while agonizing the immune response may promote tissue damage. Immune cell or tissue cell targeted drug delivery strategies may be required for effective therapeutic design in order to strike the balance between required inflammation and cytoprotection, particularly when antibiotics are not an effective option.

### Intracellularly Replicative Bacteria

#### Staphylococcus aureus

*Staphylococcus aureus* is a Gram-positive bacteria that is a facultative intracellular pathogen. The behavior of the bacteria and their cell death manipulation strategies post-engulfment or intracellular invasion differ starkly different from their activity extracellularly. In the intracellular context, they commonly replicate within the autophagosomes or cytoplasm of diverse host cell types (104). Modulation of RCD from the intracellular niche is one of the main strategies utilized by *S. aureus* against epithelial and endothelial cells that comprise pulmonary tissue and macrophages, neutrophils, and monocytes that mediate immune clearance. These strategies for intracellular RCD manipulation enable *S. aureus* to establish chronic pulmonary infections by avoiding phagocytic and efferocytic clearance and interaction with therapeutic antibiotics (105). The ability to escape the intracellular niche, and re-establish acute pyrogenic extracellular infection further exacerbates *S. aureus* infections and complicates clinical treatment strategies (105). Intracellular *staphylococci* within tissue cells, tend have a small colony morphology and downregulate batteries of genes required for virulence. Downregulated genes particularly include α-hemolysin and other secreted toxins that would induce RCD stress responses that would deny the intracellular niche (106, 107). Suppression of the agr quorum sensing system during intracellular infection contributes to induction of this phenotype by limiting secreted protein production and upregulating cell wall associated proteins (106).

Intracellular *S. aureus* must not only suppress their own cytotoxic activities, but also must subvert RCD mechanisms to restrict intracellular pathogens, such as autophagy and apoptosis, partially through the induction of anti-apoptotic and pro-survival pathways (108). A recent study by Medina et al., investigated the dynamic interactions between 16HBE14o- lung epithelial cells and intracellular *S. aureus*, up to 4 days after infection by a time-resolved analysis of both the bacteria and the host cells by mass spectrometry. Proteomic analysis revealed significant modulation of RCD regulating and effector proteins within bronchial epithelial cells by *S. aureus* up to 4 days post-infection. Specifically, internalized *S. aureus* were found to activate pathways required for the sequestration and utilization of arginine from the host cytosol (109). This metabolic activity has several effects that impede host defense via RCD, including limiting ROS production by iNOS to prevent apoptosis induction that promotes efferocytic clearance of intracellular bacteria. The deamination of sequestered arginine by *S. aureus* also releases ammonia into the cell, which increases cytosolic pH and inhibits pH gradient dependent fusion of bacteria containing endosomes with lysosomes and autophagosomes (109). Arginine starvation is known to induce autophagy, making this manipulation of RCD useful for preventing autophagic clearance of intracellular *S. aureus* (110). Other research has also shown that the induction of autophagy favors *Staphylococcus* intracellular survival (111). Lipidated autophagy-related protein (LC3B-II) conjugates are greatly enriched in epithelial cells infected with *Staphylococcus*. Inhibition of the autophagy-activating kinase 1 (ULK1) suppresses *Staphylococcus*-induced autophagy and *Staphylococcus intracellular replication* (112). *S. aureus* also is also able to escape from LC3B labeled autophagosomes, in part by impeding the maturation of autophagosomes. This is accomplished by the activation of MAPK14, which has been reported to block autophagosome maturation (113). This effect was most prominently observed in the vicinity of *S. aureus* containing autophagosomes, implying that unidentified bacterial factors are likely responsible for such activation (114). In sum, these findings provides further evidence that while intracellular *S. aureus* induces host cell autophagy pathways, it also manipulates the execution of autophagy to promote its intracellular niche.

A well-defined feature of *S. aureus* is its capacity to induce apoptosis through α-toxin activity, which is also required for phagosomal escape intracellularly (108). Medina et al., also found that host regulators of p53 apoptosis including BAG6 and DDX5 were upregulated during early infection. However, in addition to the downregulation of secreted toxins and virulence factors described previously, they also observed that intracellular *S. aureus* promoted the expression of anti-apoptotic proteins which suppressed the expression of apoptotic effectors and proteins known to correlate with the execution of apoptosis (109). Like many intracellular pathogens, the induction of cytoprotection to prevent RCD mediated restriction of the intracellular niche is an essential activity for intracellular *S. aureus.* Intracellular infection of human monocyte derived macrophages led to increased expression and stability of the key anti-apoptotic regulator MCL-1 (115). This upregulation of MCL-1 was also associated with increased secretion of interleukin-6 and activation of NfκB, both of which are pro-inflammatory, but also necessary for MCL-1 dependent cytoprotection (115). Interestingly, suppression of MCL-1 via siRNA in this system abrogated the cytoprotection and interleukin-6 secretion induced by intracellular *S. aureus* (115). Such cytoprotective activities by intracellular staphylococci may contribute to the establishment of chronic pulmonary infections *in vivo.* Further elucidation of these mechanisms could lead to promising therapeutic approaches modulating apoptotic RCD could help clinicians restrict the viability of intracellular *S. aureus*.

Summary – *S. aureus* infection and treatment are both profoundly complicated by the bacteria’s ability to act as an extracellular pyrogenic pathogen inducing RCD and its capacity to invade cells and adopt a RCD suppressive intracellular phenotype. Intracellularly, *S. aureus* has evolved the capacity to induce anti-apoptotic phenotypes that promote the maintenance of an intracellular niche. The capacity to invade immune cells, while inhibiting their effector functions and paracrine signaling, suppresses immune clearance. Their ability to invade tissue cells promotes chronic infections by shielding intracellular bacteria from patrolling immune cells, as well as endogenous and exogenous antibiotic compounds. *S. aureus-*induced suppression of phagosome and autophagosome vesicle maturation is essential for its cell invasive tropism. Therapeutic strategies restoring autophagic and phagocytic functions in host cells could significantly diminish the clinical burden of chronic *S. aureus* infections by denying the intracellular niche, as well as promoting *S. aureus* susceptibility to therapeutic antibiotics and immune clearance.

#### Mycobacterium tuberculosis

Mycobacterium, including *Mycobacterium tuberculosis*, are intracellular pathogens which form pulmonary lesions consisting of a core of necrotic cells and free extracellular bacteria (116). This necrotic core is surrounded by immune cells such as macrophages and neutrophils, which *mycobacterium* infect to enable intracellular division (116). To promote the stability of their intracellular replicative niche, *mycobacterium* have evolved host-interacting proteins that interfere with host signaling driving apoptosis (117). This inhibition of apoptosis effectively shunts infected cells toward a necrotic cell death morphology when the intracellular bacterial burden becomes too severe. This results in the expansion of the tubercular lesion and exacerbates the morbidity and mortality of mycobacterial infections by interfering with lung function. *M. tuberculosis* has struck a delicate balance between inducing the immune cell recruitment and pro-inflammatory response necessary to induce the formation of a granuloma with a necrotic core, without succumbing to the immune response and being cleared.

*Mycobacterium tuberculosis* employs components of its ESX-1 secretion system to inhibit macrophages’ ability to execute apoptosis and deny *M. tuberculosis* the intracellular replicative niche. For instance, overexpression of the EspR protein in a macrophage cell line increases intracellular *M. tuberculosis* bacterial burdens. This effect was elicited via suppression of iNOS activity and targeted inhibition of MyD88-mediated TLR signaling that promotes apoptosis and cytokine expression (118). The same research team also identified the secreted bacterial protein MptpB as a mycobacterial virulence factor similarly suppressing iNOS and apoptosis, but via the inhibition of MAPK and p53 signaling (119). This suggests that *M. tuberculosis* is under selective pressure to evolve diverse and redundant mechanisms to modulate host cell death by limiting host apoptosis to promote intracellular replication and driving cells toward a necrotic phenotype that promotes the formation of tubercular lesions.

Another mycobacterium, *M. smegmatis*, also has direct effectors that inhibit cellular apoptosis signal transduction. The overexpression of recombinant *M. smegmatis* protein AcpM, an acyl carrier protein, significantly reduces the degree of ROS production and JNK signaling in infected bone marrow-derived macrophages (BMDMs). This promotes a significant increase in the survival of infected macrophages, which contributes to the pathogenesis of mycobacterial infections by also improving the intracellular survival and replication of the pathogen. *M. tuberculosis* also expresses similar acyl carrier proteins which exert similar effects (120).

Virulent strains of *M. tuberculosis* have also long been known for their ability to inhibit apoptosis via the manipulation of host cytokine responses. For instance, the avirulent strain H37Ra was found to induce significantly more apoptosis relative to a virulent strain H37Rv (121). Later work found that H37Rv induces greater IL-10 secretion relative to the avirulent strain H37Ra. AMs infected with H37Rv are found to be secreting TNF-α in an attempt to drive apoptosis, but this is subverted by IL-10-mediated expression of TNFR2, a soluble TNF receptor which inhibits TNF signaling via direct binding (122). This activity is dependent on the endocytic uptake of mycobacterial heat shock protein 60 interacting with TLR2 (123). Another major pro-survival regulator that is driven by *M. tuberculosis* during infection is PPARγ. *M. tuberculosis* synthesized mannosylated lipoarabinomannan, has been shown to enhance PPARγ activity by activating upstream mannose receptors. This induction of PPAR activity drives the expression of the pro-survival protein MCL-1, as shown by the gene expression analysis during infection and the loss of MCL-1 expression during infection in a PPAR knockout model. The expression of MCL-1, when a phagocyte is attempting to control an intracellular infection, significantly impairs the host’s ability to restrict infection (124).

In addition to suppressing host apoptotic signaling, Mycobacteria also have several means through which they promote immune cell necrosis to expand the necrotic core of tubercular lesions. They have evolved diverse effector mechanisms to inhibit phagocyte activity in the granuloma, including through direct killing by leukocidin effectors targeting both neutrophils and macrophages. One such mechanism targeting neutrophils is the ESX-1 type VII secretion system, which induces the necrosis of neutrophils. One bacterial effector protein carrying out this induction is ESAT-6, which induces a calcium influx into the host cell that activates the protease calpain. Hyperactivity of ESAT-6 in a calcium-rich environment induces a secondary necrotic phenotype characterized by the exposure of phosphotidylserine on the outer leaflet of the cell membrane and loss of membrane permeability (125).

Utilizing its ESX-1 secretion system, *M. tuberculosis* has been shown to induce potassium efflux and calcium influx in infected macrophage cell lines. The loss of ion homeostasis subsequently drives the activation of the NLRP3 inflammasome, which results in gasdermin D (GSDMD)-mediated pyroptosis. Visualization of this effect by TIRF microscopy found the induction of pyroptosis to be dependent on bacterial proximity to the plasma membrane (126). This effect could be elicited by both extracellular and intracellular bacteria. The mechanism through which the ESX-1 secretion system disrupts membrane permeability and ion homeostasis remains poorly understood, but evidence across the field indicates that pyroptotic signaling likely contributes significantly to the formation of the necrotic core of tubercular lesions. *M. tuberculosis* also employs its type IV secretion system to inhibit uptake and phagosome maturation in neutrophils, while also driving neutrophils to induce a necrotic phenotype which promotes extracellular release of the bacteria. This enables sustained infection of other immune cells without being killed during efferocytic clearance. *M. tuberculosis* accomplishes this by inducing hyper-production of neutrophil ROS that drives necrotic cell death and through effector proteins expressed off the RD1 genomic region, such as PPE68 and RV2626c. Human primary neutrophils infected with PPE68 and RV2626c knockout *M. tuberculosis* strains more often die through apoptotic signaling, which promotes subsequent clearance *ex vivo* by uninfected human BMDMs (127). Conversely, wild-type *M. tuberculosis* is able to induce ROS-mediated necrosis in neutrophils through the activity of its ESX-1 secretion system, and subsequently infect the BMDMs in this model (128). Many other mycobacterial proteins that are structurally similar to ESAT-6 that are also secreted through the type VII secretion system seem to function in similar pathways. Such redundancies occurring through evolution strongly support the centrality of pathogen-directed cell death in the survival of mycobacterial species (127, 128).

*Mycobacterium tuberculosis* benefits from driving the pro-necroptotic signaling to push infected cells away from classical apoptosis. Similar to many other bacterial pathogens, *M. tuberculosis* produces a secreted toxin known as tuberculosis necrotizing toxin (TNT) that promotes necroptosis. However, this induction of necroptosis is not mediated by the direct formation of membrane pores; rather, TNT-mediated necroptosis is induced in macrophages by depleting NAD+ through its NAD+ glycohydrolase activity. The depletion of cellular pools of NAD+ triggers necroptosis via RIPK3- and MLKL-dependent signaling through the necrosome, without requiring the typical TNF-(or RIPK1 propagation signal for necroptosis (129). Unfortunately, the deletion or inhibition of MLKL was not found to improve disease outcomes in vivo (130). While many would argue that this result suggests that the contribution of the necroptotic pathway to the progression of M. tuberculosis infection is minimal, we would caution that this is more likely evidence of the redundant mechanisms through which mycobacterium induce necrosis in responding phagocytes.

*Mycobacterium tuberculosis* also drives the induction of necrotic cell death by antagonizing the activity of anti-necrotic/pro-survival host responses and metabolites. One example of this antagonism is the suppression of prostaglandin E2 (PGE2) synthesis by the bacterial enzyme phospholipase C (PLC). Pharmacologic inhibition of mycobacterial PLCs was found to restore the production of PGE2 and significantly inhibit the induction of necrosis in *M. tuberculosis*-infected macrophages (131). Recent data indicate that ferroptosis, is implicated in the formation of tubercular lesions. *M. tuberculosis*-induced cell death is significantly suppressed by ferroptosis inhibition and iron chelation. The role of metal homeostasis and metabolism in infectious disease, particularly with regards to RCD, requires further investigation (132).

The centrality of modulating host cell death responses to mycobacterial pathogenesis also shows the promise of host-directed therapeutic strategies in modulating cell death for the treatment of *Mycobacterium* infections. For instance, the activity of HIF-1α is critical for the host to restrict the growth of necrotic cores in tubercular lesions. As immune cells aggregate into granulomas to restrict bacterial dissemination, the middle of the granuloma becomes increasingly hypoxic. This tends to drive cells toward a necrotic phenotype, but the activity of HIF-1α antagonizes the induction of necrosis. A model of *M. avian* infection in mice lacking HIF-1α in myeloid cells rapidly formed granulomas with a necrotic core through this mechanism (133). Promoting macrophage re-programming to survive in a hypoxic environment by driving HIF-1α expression may help to restrict the expansion of necrotic cores in mycobacterial granulomas in the lung. Additionally, dexamethasone inhibits necrotic cell death by promoting the dephosphorylation of p38 MAPK. While the basic cellular phenotype characterizing this protection is the inhibition of mitochondrial outer membrane permeabilization (MOMP)-induced cell death, the underlying molecular signals mediating this inhibition of necrotic cell death remains under-characterized (134). Diverse stimuli that induce apoptosis have also been shown to promote resilience to tuberculosis infection across many diverse model systems. For instance, crude plant extracts from Rubiaceae species that are known to induce apoptosis demonstrated antimicrobial effects *in vitro* (135). However, the induction of apoptosis restricting bacterial burdens in an *in vitro* system cannot necessarily be assumed to have a therapeutic effect when translated to an *in vivo* system. Further validation and testing of host-targeted therapeutics is required.

Summary – *M. tuberculosis* deploys secreted effector proteins from their niche inside phagocytes to inhibit microbicidal functions. Inhibited pathways include MyD88/TLR pattern recognition, ROS production mediated by apoptosis, and JNK/p53-mediated apoptosis. Pathways agonized by mycobacterial effectors secreted within phagocytes include IL-10-mediated anti-inflammatory signaling, PPARγ-mediated MCL-1 expression that inhibits apoptosis, and secreted leukecidal toxins that induce diverse necrotizing RCD modalities to form the necrotic core of tubercular lesions. Host-targeted therapeutics inhibiting necrotic RCD in host phagocytes and/or agonizing apoptotic/efferocytic effector functions downstream of MyD88/TLR pattern recognition may help resolve clinically challenging multidrug-resistant tuberculosis infections.

#### Klebsiella pneumoniae

*Klebsiella pneumoniae* is a facultative intracellular pathogen. It causes complicated pulmonary infections, which are frequently antibiotic resistant. *Klebsiella* aims to promote an amenable intracellular niche by inhibiting phagocyte apoptosis, phagosome maturation, and the acceleration of bactericidal effector functions. Many phagocytes, but particularly neutrophils, will undergo apoptosis as a means of denying this niche to intracellular pathogens like *K. pneumoniae*. Recently, researchers have found that the components of the *K. pneumoniae* capsule are able to delay constitutive neutrophil apoptosis up to 2 times the normal time span, allowing for extended intracellular survival and replication. This was accomplished through bacterial inhibition of the Bax-BCL2 signaling axis, which prevented caspase-3 activation and induced anti-inflammatory IL-8 production (136). This delay in constitutive apoptosis may be critical for *Klebsiella* species to establish infections in the lung and in newly colonized metastatic infection sites after breaching the respiratory epithelial barrier.

*Klebsiella pneumoniae* also inhibits the maturation of endosomes after engulfment into phagolysosomes. These *Klebsiella*-containing vacuoles become acidified (which benefits bacterial survival), but do not contain cathepsin D protease, indicating that the bacterial- vacuoles never successfully fuse with the lysosome. This interference with lysosomal maturation prevents the efficient clearance of bacteria from the intracellular niche of macrophages, ultimately leading to the induction of programmed cell death in infected macrophages, and enabling the spread of the pathogen to new cells via apoptotic bodies or release from membrane-permeabilized cells (137).

*Klebsiella* infection has also been observed to modify the exposure of “eat me” signals to promote the longevity of cells that eventually execute apoptosis. Particularly, *Klebsiella* was observed to inhibit the exposure of phosphatidylserine by agonizing flippases that invert phosphatidylserine from the outer to the inner leaflet of the plasma membrane. This inhibition of apoptosis in neutrophils was shown to drive neutrophils toward caspase-independent cell death via the necroptosis machinery (138). Preventing the containment of cytoplasmic contents in apoptotic bodies and inhibiting debris removal via efferocytic phagocytes are other means through which *Klebsiella* maintains a suitable intracellular niche during infection.

The centrality of pyroptotic cell death as a cell autonomous response required to restrict *K. pneumoniae* pulmonary infections has been well established in the field over the past decade. For instance, researchers demonstrated that effective induction of macrophage and monocyte pyroptotic cell death through the NLRP3 inflammasome is required for restriction of *K. pneumoniae* in an *in vivo* murine model. They found that the restriction of *K. pneumoniae* infection required the activity of ASC and NLRP3, as *Nlrp3*^–/–^ *and Asc*^–/–^ mice show significantly greater mortality. Histology also demonstrates severely attenuated inflammatory response to infection in *Nlrp3*^–/–^ mice (139). However, some clinically isolated strains of *K. pneumoniae* have also demonstrated diverse means through which they subvert the host’s pyroptotic cell death response to maintain the intracellular niche and limit bactericidal effector function. For instance, one strain of *K. pneumoniae* was reported to induce high IL-1β production, leading to a pyroptotic cell death phenotype in responding macrophages. This resulted in effective bacterial clearance via hydrogen peroxide release and efficient efferocytic clearance of pyroptotic cells. However, a second strain has evolved means to limit the induction of IL-1β, which subverts the propagation of pyroptotic cell death mediated DAMP signaling that promotes immune clearance (140). This results in bacterial survival within phagocytic macrophages and ultimately permits dissemination into the host. Particularly given the antibiotic-resistant nature of *Klebsiella* spp., an improved understanding of their methods of host immune evasion is critical in developing new therapeutic approaches. Also, the possible benefit of pyroptotic cell death induction in bacteria-laden macrophages should also be explored.

Finally, *Klebsiella* promote its dissemination from the lung to metastatic infection sites by manipulating the RCD of respiratory epithelial cells required to maintain barrier integrity. One of the ways in which *K. pneumoniae* promotes the loss of barrier integrity is through the targeted disassembly of microtubules in host epithelial cells, likely promoting anoikis-mediated epithelial cell death. The bacterial gene ytfL was found to contribute to the microtubule disassembly phenotype via random screening of genomic segments trans-expressed in *E. coli*, although complete knockout of the gene did not ablate microtubule disassembly during infection (141). This suggests that other *Klebsiella* proteins contribute significantly to the induction of microtubule disassembly and anchorage dependent cell death of epithelial cells during infection. Host factors relating to microtubule disassembly such as KATNAL1 and KATNB1 contribute to this observed phenotype, indicating a probable axis of direct host-pathogen interaction at the protein level. *Klebsiella* infection of A549 cells was also shown to upregulate transcription factors, which trigger the induction of EMT. This sort of host-pathogen interaction could be critical to the observed anoikis-like cell death phenotype due to the disassembly of epithelial cell microtubules (142). However, further work is required to confirm that such microtubule dissociation by *K. pneumoniae* terminally results in anoikis cell death.

Summary – *K. pneumoniae* are obligate intracellular pathogens that reside within phagocytes post-engulfment. They hijack phagolysosomes by inhibiting proteolytic lysosomal effectors such as cathepsin D, while allowing endosome acidification. Unlike tubercular pathogens, *K. pneumoniae* has evolved to inhibit both apoptotic and necrotizing RCD pathways, which requires further study to elucidate diverse bacterial effectors. They also are capable of inhibiting efferocytic clearance of dead cells that may contain bacteria-laden vacuoles by suppressing the exposure of “eat me” signals. Given the stealthy and RCD-suppressive nature of *K. pneumoniae* infections, host-targeted therapeutics agonizing phagocyte cell death and efferocytic clearance may prove effective clinically. Such therapeutics would be highly desirable in the context of treating antibiotic-resistant strains often observed in nosocomial infections.

#### Yersinia pestis

*Yersinia pestis* is a facultative intracellular bacteria primarily infecting responding phagocytes during pneumonic infections (143, 144). To promote clearance, infected phagocytes induce pyroptotic cell death via inflammasome signaling to deny *Y. pestis* an intracellular niche and promote immune clearance via pro-inflammatory IL-1β signaling (144). At the same time, *Y. pestis* interferes with the caspase-1-mediated inflammasome signaling by inhibiting pyroptosis using diverse effector molecules targeting signal transduction steps from pathogen recognition to cytokine secretion (145). *Y. pestis* also acts to inhibit apoptotic RCD through the caspase-3/7 signaling axis to prevent cellular condensation into easily-cleared apoptotic bodies. The inhibition of these regulated mechanisms of cell death results in caspase-8 activity along with dysregulated GSDMD-mediated cell death and inflammation through non-canonical pathways that do not promote an effective immune response and enable bacterial outgrowth (146). *Y. pestis* ultimately aims to survive the process of phagocyte engulfment and escape the phagocyte in order to resume extracellular division, while subverting the pro-inflammatory process associated with phagocytic clearance (143).

Like many intracellular pathogens, *Y. pestis* utilizes a secretion system to export virulence factors into the cytoplasm of infected cells (145). The various components of this type 3 secretion system (T3SS) are identified by the host cell as PAMPs, which trigger inflammasome activation via the activity of caspase-1 when recognized. Caspase-1 activation and inflammasome activity is commonly associated with the progression of pyroptotic cell death, which in this context promotes efficient clearance of *Y. pestis* infections. However, one of the effector proteins exported via the T3SS is YopK, which prevents inflammasome activation by inhibiting the cellular recognition of the T3SS (145). The inhibition of the inflammasome and its downstream cell death pathways greatly exacerbates the severity of *Y. pestis* infection. Other Yop proteins, including YopM, have been found to inhibit caspase-1 activation as well. YopM can inhibit caspase-1 via direct binding interactions, preventing its maturation and association with the inflammasome. The inhibition of inflammasome activity through this mechanism significantly antagonizes macrophage pyroptosis and decreases host resilience to infection (147). YopM also binds to and inhibits IQGAP1, a scaffolding protein that is central to the downstream activity of caspase-1 (148). Lastly, YopM not only restricts the traditional caspase-1-NLRP3 inflammasome, it also inhibits the pyrin-activated inflammasome (149). *Yersinia* species target the activity of RhoA by inhibiting RhoA GTPases through the activity of YopE and YopT (T3SS). This activity prevents effective bacterial clearance by macrophages, by preventing RhoA signaling from modulating cytoskeletal dynamics necessary for effective phagocytosis. To mitigate the negative effects of *Yersinia* RhoA inhibition, the host activates the pyrin inflammasome which activates caspase-1 through non-pyroptotic signaling to drive additional immune cell recruitment to the infection site. YopM inhibition of caspase-1 activity also inhibits the downstream effect of pyrin-mediated inflammasomes, exacerbating the infection (149).

Gasdermin D is normally processed by inflammasome-activated caspase-1 or caspase-11, which enables it to carry out its effector functions generating pyroptotic pores in the cell membrane. However, the *Yersinia* effector protein YopJ triggers non-canonical activation of GSDMD through the function of RIPK1 and caspase-8. This is caused by YopK inhibiting the phosphorylation/activation of TAK1 and IKK, which blocks apoptosis in response to infection, and enables GSDMD-mediated cell death to predominate while other Yop proteins inhibit the activity of IL-1β and its proinflammatory function (150).

To inhibit apoptotic cell death by infected phagocytes, *Y. pestis* also produces a cell surface-bound protease known as Pla (plasminogen activator). The Pla protein inhibits Fas-mediated apoptosis by cleaving soluble and membrane bound FasL, preventing the assembly of the DISC and the activation of caspase-3 and -7 dependent apoptosis. This inhibition of apoptosis also indirectly inhibits the host immune response, by limiting the production of cytokines and the recruitment of immune cells typically coordinated by caspase-3 and -7 dependent cell death (146). This host-pathogen interaction synergizes with the T3SS to inhibit the RCD-initiated host response to both pyroptotic and apoptotic cell death, while bacteria continue to impart direct tissue damage as a result of their outgrowth in the lung (146).

Summary – *Y. pestis* are facultative intracellular pathogens that prefer to divide extracellularly but have evolved means of surviving within engulfing phagocytes, particularly macrophages. They aim to suppress apoptosis-supporting phagolysosomal clearance by subverting DISC assembly via plasminogen activation. They also antagonize the induction of inflammasome signaling driving pyroptosis and IL-1β secretion via the direct interference by Yop family proteins and other secreted effectors. The end result of *Y. pestis* RCD interference is the induction of non-canonical GSDMD-mediated necrosis, which does not produce an effective IL-1β-mediated inflammatory response required for efficient clearance and resolution. Host-targeted therapeutics driving pro-resolving apoptotic signaling in infected cells downstream of suppressed DISC assembly, or the restoration of pro-resolving pyroptotic cytokine signaling could help resolve *Y. pestis* infections. While the clinical impact of *Y. pestis* has declined in the modern age, the development of such host-directed therapies against pathogens defined as select agents remains a critical public interest.

#### Coxiella Burnettii

*Coxiella burnetii* is an obligate intracellular pathogen that is the etiological agent of Q-fever (151). It primarily infects host phagocytes by residing in bacteria-laden parasitophorous vacuoles, relying on its anti-apoptotic activity effected through its Dot/Icm Type IV secretion system (T4SS) to maintain its replicative niche in the host (151, 152). Even during TNF-α treatment to provide a strong induction of extrinsic apoptosis, *C. burnettii* infection was observed to offer strong protection from pro-apoptotic signaling. *C. burnettii*-infected cells were observed to upregulate expression of anti-apoptotic effectors such as A1/Bfl-1 and c-IAP2 in THP-1 cells and non-human primate primary AMs. *C. burnettii*-infected cells are often characterized by stark reductions in caspase proteolytic activity with increased expression of pro-survival mediators (153). One of the many anti-apoptotic effector proteins of *C. burnetii*, is the protein AnkG. Studies ectopically expressing AnkG in CHO cells found that AnkG initially associates with the mitochondria before directly binding with p32 during early apoptotic signaling. The direct binding of AnkG to p32 results in their colocalization in the nucleus where p32’s transcriptional regulatory activity is restrained (154). Ectopic expression of *C. burnetii* AnkG in *Legionella pneumophila* was also shown to suppress pathogen-induced apoptosis in DCs, which are normally highly restrictive host cells for these intracellular bacteria (154). Recent work has also shown that AnkG requires association with both p32 and the nuclear localization protein importin α1 to carry out its anti-apoptotic function (155).

While AnkG is the most studied anti-apoptotic component of the *C. burnetii* T4SS, it is not singly responsible for carrying out the bacteria’s anti-apoptotic effector functions. Ectopic expression of two other T4SS products from *C. burnetti*, CaeA and CaeB, in CHO cells were also found to significantly inhibit intrinsic apoptosis signal transduction. CaeA was found to localize to the nucleus, while CaeB was found to localize to mitochondria and was a more effective inhibitor of apoptosis. Cells overexpressing CaeB have a significant reduction in PARP cleavage downstream of Bax activation (156). *C. burnetii* also effects its anti-apoptotic functions through interference with vesicle trafficking and the autophagic machinery. Beclin-1 (necessary for regulated maturation of the autophagosome) and BCL-2 are both recruited to *C. burnetii-*containing vacuoles and augment the replicative capacity of the bacteria by inhibiting RCD and autophagic recycling (157).

*Coxiella burnettii* also has been found to manipulate the regulation of host regulatory kinases to inhibit macrophage apoptosis. For instance, *C. burnettii* agonizes cAMP-dependent protein kinase (PKA). Studies found that pharmacologic inhibition of PKA in THP-1 cells and human AMs resulted in significantly more apoptotic cell death than untreated cells. They found that PKA propagates anti-apoptotic/pro-survival signals in the infected macrophage via phosphorylation and inactivation of Bad. *C. burnettii* was observed to direct the localization of Bad to bacteria-laden vacuoles to carry out this kinase activity (158). Avirulent *C. burnetii* was also found to inhibit caspase-3 cleavage while agonizing pro-survival MAPK p38, Erk, and PI3k signaling. Caspase-3 inhibition by *Coxiella* is effected through the activity of the pro-survival protein MCL-1, which is stabilized by Erk1 phosphorylation. Knockout studies found that the T4SS in *Coxiella* was necessary for this inhibition (159).

Summary – *C. brunettii* are obligate intracellular pathogens that reside within host phagocytes in bacteria-laden vacuoles. From these vacuoles, *C. brunettii* secrete a diverse range of anti-apoptotic effectors designed to prevent the appropriate induction and execution of apoptosis due to phagocyte pattern recognition of bacteria-laden vacuoles. Host-targeted therapeutic strategies aimed at restoring apoptosis in infected phagocytes to deny *C. brunettii* its intracellular niche could be useful in treating chronic Q-fever in patients at risk of acute infection.

#### Legionella pneumophila

*Legionella pneumophila* is an intracellular Gram-negative bacteria that infects the lungs and causes a severe pneumonia known as Legionnaires disease. Its natural hosts are free-living freshwater protozoa; however, aerosolizations of contaminated water supplies enable the bacteria to infect the human lung (160). AMs are the primary site of bacterial replication but neutrophils can also be infected (161, 162). Upon infection, *Legionella* replicates within a *Legionella*-containing vacuole (LCV). There is also evidence that it can infect lung epithelial cells, although the formation of a replicative vacuole is distinct (163). *L. pneumophila* has a T4SS, which is essential for bacterial virulence. Virulence factors of *Legionella* control many aspects of the cellular machinery including cell death pathways (164). Cells infected with *L. pneumophila* undergo a variety of types of RCD (165). Much of this is dependent on the cell type, and to some extent the strain of *L. pneumophila*.

Apoptosis is triggered in many myeloid cell types, including DCs and macrophages. Interestingly DCs undergo rapid apoptosis upon infection with *L. pneumophila*, while macrophages are relatively resistant to infection-induced apoptosis (166). Suppression of apoptosis in DCs allows for the replication of bacteria. *L. pneumophila* uses several effector molecules and strategies to avoid undergoing apoptosis upon infection. This includes the bacterial protein SidF, which interferes with the pro-apoptotic proteins BNIP3 and Bcl-rambo (167). In addition, LegK1 and LnaB increase activation of NF-b, which induces a pro-survival/anti-apoptotic phenotype. When apoptosis in suppressed even further in myeloid cells there is an increase in pulmonary inflammation, which suggests that the regulation of apoptosis is important for decreasing inflammation in the host (168).

While *L. pneumophila* has many mechanisms to decrease apoptosis, other types of cell death are increased upon infection. This includes pyroptosis, necrosis, and autophagic cell death (169, 170). Pyroptosis is triggered by both a flagellin-dependent and flagellin-independent mechanism (166, 171). Flagellin-dependent signaling through the NAIP5/NLRC4 inflammasome causes pyroptosis in macrophages from some mouse lines. This is dependent on the T4SS. Pyroptosis induced by caspase-11 signaling is also dependent on the T4SS, but independent of flagellin. Autophagy also requires virulence factors secreted by the T4SS to be induced in macrophages following infection. There is also evidence in human monocyte cell lines and primary mouse macrophages that cathepsin-dependent necrosis can be triggered through the recognition of RpsL (172).

Summary – Similar to many intracellular pathogens, *L. pneumophila* uses a variety of mechanisms to suppress apoptosis, and thus insure a niche for replication. However, the mechanisms that *L. pneumophila* takes to survive are counteracted by the host response. Given that *Legionella* is a relatively new human pathogen, the interactions with the host may still be evolving.

## Discussion

In this review, we have summarized the recent studies that have shown the impact that cell death-mediated host-pathogen interactions on pulmonary infections. We suggest that cell death inhibition, induction, and recovery function synchronously to maintain host homeostasis. Having aggregated data from diverse research teams regarding the targeted manipulation of host RCD by pulmonary bacterial pathogens, several clear trends and functional groupings can be identified. It is clear that the overall strategies of intracellular and extracellular pathogens vary greatly regarding how they use host cell death programs to promote the establishment and maintenance of infection.

Generally, extracellular pathogens are primarily agonizers of RCD, triggering the induction of regulated host-programmed cell death via direct cytocidal effectors. While many extracellular pathogens also induce apoptotic cell death, the secretion of such direct effectors (including pore-forming toxins, exotoxins, and ROS), likely induces membrane permeabilization in apoptotic cells as well (often referred to as secondary necrosis). The less discriminating nature of extracellular pathogens to target cell types is due to their mechanism of action being effected through direct physical disruption or destruction of cellular organelles and proteins, rather than targeted inhibition via binding. As such, extracellular pulmonary pathogens impact respiratory epithelial cell mucociliary defenses and barrier integrity, along with immune cell effector functions and maintenance using the same biologic activities. The direct and less discriminate damage imparted by extracellular pathogens establishes a pulmonary niche for the bacteria that is analogous to a cellular war zone bathed in pro-inflammatory mediators in the form of cellular debris, cytoplasmic nutrients, and PAMPs. This environment is uniquely conducive to bacterial dissemination to distal infection sites due to bacterial translocation from the lung to the bloodstream.

Antibiotics are the primary intervention for such bacterial infections. However, understanding that extracellular pulmonary pathogens rely on the induction of such membrane-permeabilizing RCD modalities for their pathogenesis allows us to consider novel therapeutic approaches that target the host to treat pneumonia. The inhibition of membrane-permeabilizing cell death that these bacteria rely on to form an amenable pulmonary niche could hold significant advantages relative to pathogen-targeted therapeutic strategies. Firstly, by targeting host factors to augment tolerance to infection, pathogens will be less likely to escape the therapy by evolving resistance to pathogen-targeted therapeutics, which rely on binding inhibition of specific bacterial targets. Improving the host response to infection will also provide clinicians with alternative approaches for treating antibiotic-resistant infections under the same rationale. Understanding that diverse extracellular pathogens induce the same forms of cell death using similarly evolved mechanisms of action also allows us to consider how one might develop a class of host-directed therapeutics targeting a range of extracellular pathogens. Compounds and gene therapies limiting the induction of membrane-permeabilizing cell deaths via secondary necrosis, pyroptosis, necroptosis, and ferroptosis may inhibit the formation of pulmonary niches conducive to the progression of extracellular bacterial pneumonia. The simultaneous inhibition of several cell death pathways may be required to design effective therapeutics, as inhibiting one form of membrane-permeabilizing cell death may simply push cells toward another membrane-permeabilizing cell death modality. Such approaches to controlling the inflammatory environment by limiting environmental DAMPs would also avoid key pitfalls of many host-targeted therapies such as corticosteroids, which directly suppress immune effector functions to limit immunopathology. Inhibition of membrane-permeabilizing RCD, the promotion of membrane integral cell death, the promotion of barrier integrity or epithelial cell autonomous defenses, and many other host factors should be considered for investigation in designing host-directed therapeutic approaches leveraging cell death.

With regards to intracellular bacterial pathogens’ manipulation of RCD, they generally aim to promote an amenable intracellular niche for bacterial replication within phagocytic cells or cells that may endocytose pathogens. This typically requires targeted inhibition of apoptotic signal transduction, but the mechanisms employed by specific bacteria to accomplish such inhibition varies widely. With this in mind, intracellular pathogens can also be further divided into two functional categories. One class of intracellular pathogens are pro-inflammatory and generally present an acute pneumonia with necrotic cell phenotypes driven in responding immune cells. Such pathogens will often rely on the induction of necrosis in bacteria-burdened cells, which both promotes the release of bacteria to infect new cells and drives further immune cell recruitment and tissue damage. Often, these bacteria will utilize the immunopathology-induced damage to the lung epithelial barrier to cross over into the bloodstream and colonize metastatic infection sites, which contributes significantly to morbidity and mortality. Such pathogens, including *K. pneumoniae* and *M. tuberculosis*, are serious clinical threats, which are often greatly complicated by the existence of multiple drug resistant serotypes. Designing host-targeted therapeutic strategies against these bacteria might rely on the induction of non-permeabilizing cell death to trap bacteria in apoptotic bodies or the promotion of “eat me” signals on apoptotic cells to more efficiently clear them via efferocytosis. Pharmacologic suppression of membrane-permeabilizing cell death during such infections also might promote non-permeabilizing RCD that restricts bacterial growth. However, it is important to note the overlapping complexities of RCD may require combinatorial inhibition of permeabilizing cell death pathways to elicit therapeutic effects. Further work determining underlying mechanisms through which intracellular bacteria manipulate pro-inflammatory cell death is required, alongside the design and testing of novel therapeutic strategies modulating cell death.

The other classes of intracellular pathogens are primarily focused on suppressing pattern recognition signaling, inflammation, and immune effector functions associated with the induction of both membrane-permeable and non-permeable RCD. They also tend to drive the upregulation of host factors promoting cellular survival and replication, which would normally be rapidly inactivated upon sensing an intracellular pathogen. These intracellular bacteria tend to establish chronic infections and rely on stealth to maintain an amenable pulmonary niche for replication. For instance, *C. brunettii* infection can develop into chronic Q-fever months or even years after initial infection (173). These infections can often persist, even after aggressive treatment with broad-spectrum antibiotics (173). Difficulty clearing such infections, even with the application of typical antibiotic therapies, lends support to the hypothesis that pharmacologic control of RCD dynamics could be used to promote clearance. Applying therapeutic compounds to promote the induction of apoptotic death or subvert pathogen blockade of RCD signals should be investigated in this context. Some researchers have investigated such approaches *in vitro*, but research aimed at translating positive findings inducing cell death to restrict bacterial pneumonia is required (135). Such approaches could be used in conjunction with traditional antibiotic therapeutics to eliminate stealthy and stubborn pulmonary infections by direct killing and denial of an amenable intracellular niche.

### Conclusion and Open Questions

Pulmonary bacterial infection triggers diverse host cell responses that include activation of cell death and/or survival signaling, inflammatory responses, and the immune system. Cell death is now recognized by the work of multiple labs as an important, druggable target which controls the degree of inflammatory injury in many pathologies, including ischemia–reperfusion injuries (heart, brain, kidney, and liver), brain trauma, eye diseases, and pulmonary bacterial infections (174). Because barrier disruption not only depends on cell death but also on the capacity of the remaining cells to proliferate and keep the barrier sealed, it is important to also study whether different forms of necrosis regulate key barrier-related properties of neighboring pulmonary epithelial cells such as proliferation, migration, and adhesion. The host’s regulation and bacterial manipulation of RCD is a critical determinant of outcomes during bacterial pneumonia. The literature cited in this article shows that pulmonary bacteria can induce a vicious circle of cell death, pore-forming toxin release, activation of immune-cell, and the release of death-inducing cytokines and toxins that may fuel prolonged non-resolving inflammatory responses and contribute to the pathogenesis of chronic lung inflammatory diseases.

Cell death is now established as an important strategy to control bacterial infection and/or promote host tolerance, but many questions remain to be addressed. Is cell death a primary driver or secondary mechanism manipulated by bacterial pathogens to successfully establish within the host? Can targeting cell death-related proteins be an effective strategy as anti-bacterial therapeutics? What is the relative contribution of cell death-dependent functions of bacteria-infected phagocytes in inflammation? How does cell death contribute to the initiation, amplification, and chronicity of lung inflammation? On a mechanistic level, what are the different bacterial effectors involved with cell death manipulation and why do some bacteria selectively induce membrane non-permeabilizing death pathways? Which therapeutic strategy would be the most effective against cell death-associated lung injury in clinical settings? Can we predict and interfere with host outcomes whether a species of bacteria is more or less prone to induce cell death? How does infection with multiple pathogens influence RCD? We posit that current progress in the field suggests that therapeutics modulating host RCD signaling to treat infectious disease may soon be a reality, but further investigations into pulmonary host-pathogen interactions governing cell death are required in the interim to meet this goal.

## Author Contributions

EF, NL, and AJ designed all the research, wrote the manuscript, and contributed data to the table. EF finalized the table. EF and NL made the figure.

## Conflict of Interest

The authors declare that the research was conducted in the absence of any commercial or financial relationships that could be construed as a potential conflict of interest.
